# Associations between Profiles of Self-Esteem and Achievement Goals and the Protection of Self-Worth in University Students

**DOI:** 10.3390/ijerph16122218

**Published:** 2019-06-23

**Authors:** María del Mar Ferradás, Carlos Freire, José Carlos Núñez, Bibiana Regueiro

**Affiliations:** 1 Department of Psychology, University of A Coruña, 15071 A Coruña, Spain; carlos.freire.rodriguez@udc.es (C.F.); bibiana.regueiro@udc.es (B.R.); 2 Faculty of Psychology, University of Oviedo, 33003 Oviedo, Asturias, Spain; jcarlosn@uniovi.es

**Keywords:** self-handicapping, defensive pessimism, self-esteem, achievement goals, motivational profiles

## Abstract

The high demands of academia and the fear of failure lead some university students to prioritize defending their personal worth through the use of complex strategies such as self-handicapping or defensive pessimism. Adopting a person-centered approach, this study established two objectives: First, to analyze the conformation of different motivational profiles based on the combination of self-esteem and achievement goals (learning, performance approach, and performance avoidance); and second, to determine if the identified profiles differ from one another in the use of self-handicapping and defensive pessimism. A total of 1028 university students participated in the research. Four motivational profiles were obtained: (a) High self-esteem, low learning goals, high performance approach goals, and high performance avoidance goals; (b) high self-esteem, high learning goals, low performance approach goals, and low performance avoidance goals; (c) low self-esteem, low learning goals, high performance approach goals, and high performance avoidance goals; and (d) low self-esteem, high learning goals, high performance approach goals, and medium performance avoidance goals. Profiles (c) and (d) were significantly related to self-handicapping and defensive pessimism, respectively. These results suggest that students with low self-esteem are more vulnerable to self-protection strategies. Additionally, under self-handicapping and defensive pessimism, the achievement goals are slightly different.

## 1. Introduction

In line with the challenges posed by the current social emphasis on self-directed and lifelong learning [1,2], university students are subject to high academic requirements. Some students manage this challenge with a clear orientation to success, indicating a high degree of motivation and enthusiasm for learning [3]. For other students, this demanding context poses a significant threat because they perceive academic failure as evidence of low self-worth [4]. Under these conditions, strategies such as self-handicapping or defensive pessimism are powerful incentives to protect their feelings of competence.

In this study, the role of self-esteem and achievement goals as motivational determinants of self-handicapping and defensive pessimism strategies is analyzed. Researchers have analyzed the role played by both motivational factors individually, but not in a combined manner. Adopting a personal-centered approach, we sought to identify different students’ motivational profiles based on their level of self-esteem and the use of three types of achievement goals (learning, performance avoidance, and performance approach). Specifically, the method used is intended to determine which student profiles are motivationally more vulnerable to self-worth protection strategies.

### 1.1. Self-Protection Strategies: Self-Handicapping and Defensive Pessimism

Self-handicapping constitutes an anticipatory mechanism through which the student sabotages her or his own probabilities of success by creating an obstacle, real or fictitious, that serves as an alibi against an expected failure [5]. This strategy, therefore, outsources the reasons for a hypothetical low performance to focus attention on the handicap rather than on personal incompetence. Self-handicapping can be effective in the short term because it serves to protect the student’s sense of self-worth [6]; however, recurrent use often leads to academic damage (e.g., low performance, dropout) [7], which result in fractured feelings of personal worth [8].

Similarly, through the strategy of defensive pessimism, the student, despite his or her previously good academic record, establishes excessively low expectations of achievement that he or she perceives as safe in response to the anxiety generated by a failure [9]. Paradoxically, the low expectations are often the prelude to hard, generally successful work and aimed at avoiding hypothetical failure [10]. Therefore, in the short term, defensive pessimism usually contributes to academic success [11]; however, in the long run, it entails significant detriments to emotional health [12].

One of the topics that has raised the most interest in the research on self-handicapping and defensive pessimism is determining the motivational factors underlying these strategies. Among these factors, according to the literature, are self-esteem and achievement goals.

### 1.2. Self-Esteem and Self-Protection Strategies

Self-esteem is a positive or negative attitude that reflects the degree to which the person feels self-appreciation, -value, and -satisfaction [13,14], significantly affecting the involvement and achievement of students [15,16]. The findings of the studies that have analyzed the relationship between self-esteem and self-protection strategies have been inconsistent. Some works have demonstrated a negative relationship between self-handicapping and self-esteem [17,18], whereas others have asserted that self-handicapping is positively related to self-esteem [19,20]. Another suggestion was that people with low self-esteem would use self-handicapping to protect their image and people with high self-esteem to enhance their image [21]. Along the same lines, some studies have concluded that defensive pessimism underlies negative self-reported thoughts [22,23]. However, Ferradás, Freire, Valle, and Regueiro [24] identified profiles of defensive pessimistic university students with low and high self-esteem. Consistent with this ambiguity, Yamawaki, Tschanz, and Feick [25] raised the possibility that defensive pessimistic students show high self-esteem in some situations and low self-esteem in other situations.

### 1.3. Achievement Goals and Self-Protection Strategies

Goals are generally conceptualized as cognitive representations of specific desired outcomes or end states that the individual is committed to either approach or avoid; therefore, they guide behaviors, emotions, and cognitions [26,27]. Accordingly, from the perspective of the achievement goal theory [28,29], the achievement goals represent a future-focused cognitive representation of the results or states that govern the behaviors of involvement and achievement of students in academic tasks [30].

Central to achievement goal theory is the definition of competence; that is, the standard or referent used to determine if an individual is doing well or poorly [31]. Traditionally, researchers have distinguished two major opposite views of competence in achievement situations, and such a distinction has led to differentiation between learning goals and performance goals. Learning goals characterize students who believe that competence is a malleable quality that can be developed and cultivated. As a result, this type of student is fundamentally focused on their own competence [32]; thus, they are oriented toward achieving a broad range of intrapersonal standards [33], for example, fulfilling their interest and curiosity, improving their competencies and doing better than one has done in past situations, learning as much as possible, mastering the requirements of the task, and coping with personal challenging activities.

By contrast, students who adopt performance goals believe that competence is a fixed trait (something that you have or you do not have); thus, their principal focus is on comparing their competence with others [34]. Performance goals can be subdivided into performance avoidance goals and performance approach goals. Students pursuing performance avoidance goals seek to avoid doing worse than others (appearance criterion), avoid being negatively judged by others (normative criterion), or both (evaluative criterion). Students with performance approach goals are oriented toward outperforming other students (normative criterion), demonstrating competence to others (appearance criterion), or both (evaluative criterion) [33].

In general, self-handicapping and defensive pessimism are associated with performance goals. In this sense, some studies have affirmed that both strategies are fundamentally motivated by performance avoidance goals [17,35,36]. However, other works have concluded that performance approach goals also constitute a relevant antecedent of self-handicapping and defensive pessimism [37,38,39,40,41]. Regarding learning goals, the adoption of this type of goal has been observed to reduce the tendency to self-handicap [17,42]. Evidence has also been presented that learning goals are negatively related to defensive pessimism [25,37], although some works have found some combined use of learning and performance goals in defensive pessimistic students [38,43].

### 1.4. This Study

Based on our review of the literature, self-esteem and achievement goals emerge as powerful motivational determinants of self-handicapping and defensive pessimism strategies in academic settings. To date, the work carried out in this field has focused on analyzing the role played by self-esteem and achievement goals individually. However, and notably, academic motivation, as suggested by Pintrich and De Groot [44], is the result of combining three motivational components: A value component that includes students’ goals in academic tasks (e.g., achievement goals); an expectancy component, comprising students’ perceptions, judgements, and feelings about themselves (e.g., self-esteem, self-efficacy); and an affective component, involving students’ emotional reactions to the academic tasks (e.g., satisfaction, anxiety, fear of failure).

Accordingly, the approach adopted by researchers (a variable-centered approach, i.e., an approach focused on the effect that each variable, or at least each motivational component, exercises independently on other variables) had not accurately described this complex motivational reality. Therefore, studies that adopt a person-centered approach are needed, because they would make it possible to know how the student integrates different variables into an individual motivational profile [45]. Unlike variable-centered approaches, which assume that the population is homogeneous with respect to how the predictors operate on the outcomes, person-centered approaches assume that the population is heterogeneous with respect to how the predictors operate on the outcomes; thus, they allow the identification of subtypes of individuals who share particular attributes (having similar values in a set of variables) that differ from other individuals [46]. In other words, person-centered approaches identify “how individuals may be classified based on a set of variables, thus generating typologies of people according to their shared responses across multiple markers” [47] (p. 289), and, as a result, allow for the identification of specific combinations of variables that occur naturally in a sample. In this study, profile typologies based on different functioning levels of achievement goals (value component of academic motivation) and self-esteem (expectancy component of academic motivation) markers might be useful for identifying subgroups of students that might be particularly susceptible to self-handicapping and/or defensive pessimism strategies.

In the academic field, a relatively extensive body of research has found that students, rather than adopting a single type of goals, tend to adopt several goals [48,49]. This person-centered approach has revealed a wide range of possible combinations (i.e., profiles) of achievement goals in the university context, based on the conjugation of learning goals and performance goals [50,51]. The identified profiles of achievement goals have been associated with different cognitive, emotional, and achievement outcomes. In particular, those groups of multiple goals that include high levels of learning goals (regardless of the level of each performance goals) present highly adaptive academic consequences, such as high engagement and performance. By contrast, those profiles of multiple goals that combine high levels of performance goals with low learning goals, are observed to be more vulnerable, and the students with those profiles have displayed more anxiety and lower control beliefs about their academic achievement [49,52,53,54,55].

However, in our review of the literature, no precedents have accounted for self-esteem in the study of students’ multiple goal profiles. This approach seems pertinent because, in motivational terms, defined achievement goals will be of little use if the student feels no self-worth. Accordingly, our study attempts to identify motivational profiles of university students based on the combination of self-esteem and the main achievement goals (i.e., learning, performance avoidance, and performance approach).

In general, the studies that have analyzed (from a variable-centered approach) the relationship between self-esteem and achievement goals have linked self-esteem positively with learning goals and negatively with performance avoidance goals [56,57,58,59]. The self-esteem–performance approach goals relationship is even more controversial because positive [57,59] and negative [58,60] evidence has been presented regarding relationship between these two elements. Furthermore, the few studies that have considered the multiple goal perspective confirmed that self-esteem is higher in those profiles that excel due to high orientation to learning goals, and those that combine learning goals and performance approach goals. By contrast, the lowest self-esteem would be found in those profiles with a predominance of performance avoidance goals [61,62].

### 1.5. Aims of the Study

The present study had two main objectives: (1) To analyze the degree to which different motivational profiles are conformed to, based on the combination of self-esteem and achievement goals; and (2) to determine if the identified profiles differ in the use of self-handicapping and defensive pessimism.

Regarding the first objective, the literature offers no precedent about the existence of combined profiles of self-esteem and achievement goals. However, we have expectations regarding some results according to the revised works on students’ multiple goal profiles and those studies that have analyzed the relationship between self-esteem and achievement goals. As aforementioned, the research on achievement goals has identified a broad spectrum of multiple goal profiles. In this sense, notable differences have been observed across the studies regarding how and how many profiles endorse, with findings ranging from four to seven profiles [49,53,54,55,63]. Based on the most common profiles identified by the multiple goal literature, we expected to identify four profiles in our study: (a) High in all achievement goals (learning, performance avoidance, and performance approach); (b) high learning goals and low performance goals (both avoidance and approach); (c) high learning goals, low performance avoidance goals, and high performance approach goals; and (d) low learning goals, high performance avoidance goals, and high performance approach goals. Other students’ multiple goal profiles (e.g., a profile of high learning goals, high performance avoidance goals, and low performance approach goals; a profile of low learning goals, low performance avoidance goals, and high performance approach goals; a profile of low learning goals, high performance avoidance goals, and low performance approach goals) are not expected, given their infrequent occurrence across the studies that have adopted a person-centered approach [64].

**Figure 1:**
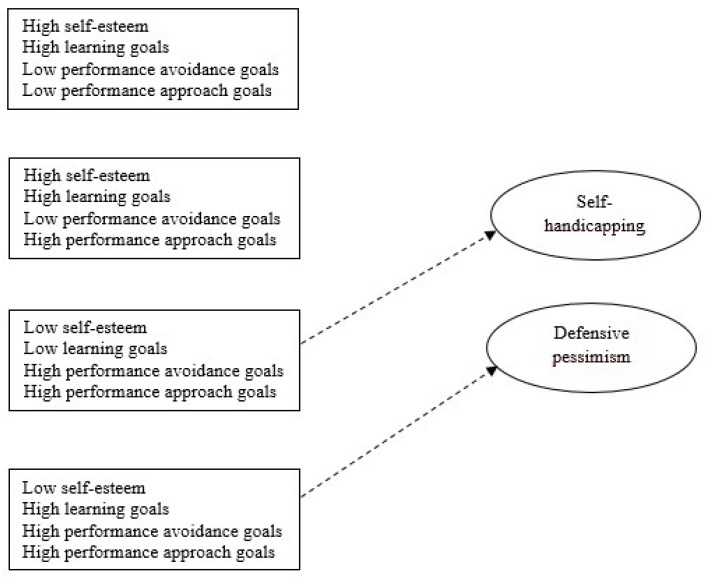
Hypothesized motivational profiles and expected associations to self-protection strategies.

Likewise, based on the revised studies that have analyzed the relationship between self-esteem and multiple goal profiles by using a variable-centered approach, we plausibly hypothesized that the profiles of students salient in performance avoidance goals would show low levels of self-esteem. In effect, the students who adopt performance avoidance goals typically feel that they are unworthy people [4,65]. By contrast, the students who exhibit a predominance of learning goals and performance approach goals have been observed to feel more self-confident, either by attempting to improve her or his ability or by demonstrating their competence to others [31,34]. Therefore, we expected that the profile that combines high learning goals with low levels of both performance goals, and the profile that comprises high learning goals, low performance avoidance goals, and high performance approach goals would show high self-esteem. The graphic representation of the hypothesized motivational profiles is shown in Figure 1.

As a second objective, we aimed to determine which motivational profiles relate to a greater extent to self-handicapping and defensive pessimism. Regarding self-handicapping, some of the reviewed studies have found that these strategies are more prevalent in students with profiles that combine a high use of the performance avoidance and performance approach goals [38,40,41]. Additionally, learning goals were observed to buffer the positive relationship between performance goals and self-handicapping [41,66].

In this study, the distinction between behavioral and claimed self-handicapping was considered because this typological taxonomy is well established in the field [67]. Behavioral self-handicapping refers to any direct action (e.g., procrastinating, over involvement in multiple tasks simultaneously) undertaken to serve the purpose of obstruction. Claimed self-handicapping entails the verbalization of an impediment (e.g., fatigue) without performing a self-limiting external behavior. That distinction is important because behavioral self-handicapping was observed to be more maladaptive than claimed self-handicapping in academic settings [68] because the former always compromises achievement, whereas claimed self-handicapping does not necessarily compromise achievement [69]. According to our review of the literature, the only precedent has studied the relationship between multiple goal profiles and both types of self-handicapping [38] and found that the students who combine low learning goals with high performance avoidance goals and high performance approach goals tend to use claimed and behavioral self-handicapping to a high degree. Based on this finding, we expected that the hypothesized profile of low learning goals, high performance avoidance goals, and high performance approach goals would use both types of self-handicapping (behavioral and claimed) to a greater degree.

Notably, although the relationship between self-esteem and self-handicapping has remained controversial [17,20], a plausible consideration is that self-handicapping would be less likely in people with high self-esteem. As Berglas and Jones [70] stated, no one would expect that “people who know they have the talent and resources to master life’s challenges (…) to hide behind the attributional shield of self-handicapping” (p. 406). Accordingly, we hypothesized that the motivational profile of low self-esteem, low learning goals, high performance avoidance goals, and high performance approach goals would be the group that would more frequently resort to behavioral and claimed self-handicapping (Figure 1).

Regarding defensive pessimism, this self-protective strategy is based on a double intention to succeed and to avoid failure [9,37]. Accordingly, some of the revised works have found that defensive pessimism is positively related to both performance avoidance and performance approach goals [25,37]. Similarly, other studies have found a greater use of defensive pessimism in students who adopt both performance goals and learning goals [38,43]. Likewise, a negative relationship between defensive pessimism and self-esteem has been demonstrated [22,23]. Based on these considerations, we hypothesized that the motivational profile of low self-esteem and high use of the three achievement goals (learning, performance avoidance, and performance approach) would use defensive pessimism to a greater degree than the other profiles (Figure 1).

## 2. Materials and Methods

### 2.1. Participants and Procedure

A total of 1087 university students from University of A Coruña (Spain) were selected by convenience sampling to participate in the study. Of these students, 56 cases were excluded because they presented a high rate of missing data (higher than 20%). Another 27 cases showed a lower rate of missing data, which were treated using the full information maximum likelihood (FIML) method through the MPlus 7.11 program [71]. In addition, three other cases presented as outliers (Mahalanobis distance method) when exceeding the critical value χ^²^ = 5.5 (gl = 7, *p* < 0.001); thus, they were also discarded.

The final composition of the participating sample was 1028 students aged 18 to 25 years (*M_age_* = 21.36, *SD_age_* = 3.81): 887 (86.3%) were women and 141 (13.7%) were men. Of the participants, 69.9% were enrolled in health sciences degrees (nursing, physiotherapy, podiatry) and 31.1% in education sciences (early childhood education, primary education, social education, and speech therapy). Regarding grade levels, 37.2%, 32.5%, and 30.3% of the students were in their first, second, and third years, respectively.

The study was conducted according to the guidelines of the Ethics Committee at the University of A Coruña (ethical code: 03/04/2018) [72], with written informed consent from all participants, as established in the Helsinki Declaration. The data for the research were collected in the classrooms where the participants were taught, within the class schedule, and in a single session without a time limit. The participants were asked for their impartial collaboration, and the researchers indicated the objectives of the research and guaranteed the anonymity and confidentiality of participants’ data.

### 2.2. Instruments

Self-esteem. The validated version of the Rosenberg self-esteem scale in Spanish was used [73]. The instrument comprises 10 items that measure feelings of self-appreciation and self-acceptance. Five items are positively worded (e.g., “In general, I am satisfied with myself”) and the other five are negatively worded (e.g., “All in all, I am inclined to feel that I am a failure”). Participants’ responses were evaluated using a Likert scale (1 = in total disagreement to 5 = in total agreement). The reliability obtained by the scale in this study was α = 0.88.

Achievement goals. The Spanish adaptation of the Skaalvik goal orientation scale [74] was used to analyze the following goals: Learning goals (six items; e.g., “It’s important for me to learn new things in class;” α = 0.79) are conceptualized in terms of task orientation, that is, the students’ desire to learn and gain new knowledge [33]; performance approach goals (five items, e.g., “I try to get better grades than my classmates;” α = 0.85) represent the normative criterion (i.e., demonstrate higher abilities than others) of these goals [33]; and performance avoidance goals (six items; e.g., “When I answer incorrectly in class, I worry about what my classmates think of me,” α = 0.80) represent the appearance criterion (i.e., avoid being negatively judged by others) of these goals [33]. Participants’ responses were recorded on a Likert scale (1 = never to 5 = always).

Self-handicapping. To evaluate self-handicapping, the Spanish adaptation [38] of the self-handicapping scale [75] was used. The instrument provides two types of self-handicapping: Behavioral self-handicapping, which evaluates active handicaps (nine items; e.g., “I tend not to attempt tasks; that way I have an excuse if I don’t do as well as expected;” α = 0.84), and claimed self-handicapping, which measures verbal handicaps (16 items; e.g., “I tell others that I am more exhausted than I really am when I have to do homework or exams, so if I don’t do as well as expected, I can say that that is the reason;” α = 0.90).

Defensive-pessimism. Defensive pessimism was evaluated using the defensive pessimism questionnaire [76], which comprises 12 items (e.g., “Thinking about what can go wrong helps me prepare”), whose reliability in our study was α = 0.89. For both instruments, participants’ responses were recorded on a Likert scale (1 = never to 5 = always).

### 2.3. Data Analysis

To determine the latent categorical variables that allow for grouping the participants in classes (profiles) according to their self-esteem characteristics and the three types of achievement goals (learning, performance approach, and performance avoidance), a latent profile analysis (LPA) was performed [77]. Using the MPlus program, version 7.11 [71], we determined, from among a finite set of models, which model best fit the data, adding successive latent classes to the target model.

The optimal number of classes was determined by considering the Akaike information criterion (AIC), the Schwarz Bayesian information criterion (BIC), the BIC adjusted for the sample size (SSA-BIC), the adjusted Lo–Mendell–Rubin [78] maximum likelihood ratio test (LMRT), the parametric bootstrap likelihood ratio test (PBLRT), the size of the sample for each subgroup, and the multivariate adjustment tests for asymmetry and kurtosis. The *p* value associated with the LMRT and the PBLRT indicates whether the solution with more (*p* < 0.05) or fewer classes (*p* > 0.05) is the solution that best fits the data. The AIC, BIC, and SSA-BIC indices have a descriptive character, with the lowest values indicating a better model fit. These criteria should complement the information provided by the LMRT and the PBLRT, but in no case should it be replaced, and the latter are the criteria that allow a final decision to be made. Furthermore, classes that contain less than 5% of the sample are considered spurious, a condition indicative of the excessive extraction of profiles [79]. Likewise, we calculated a posteriori probabilities and the entropy statistic to determine the classifying accuracy of the selected model. The value of the entropy oscillates between zero and one, and the values closest to one represent the greatest classifying accuracy.

Finally, a MANOVA was performed to determine the differences between the profiles of self-esteem and achievement goals (criterion variable) in the use of self-handicapping and defensive pessimism (dependent variables). Therefore, the probabilities of the participants belonging to the different classes of the selected model were saved and then used in the MANOVA analysis. The effect size was determined by the partial eta squared and *d* statistics [80]: Null effect: η_p_^2^ < 0.01 (*d* < 0.09); small: η_p_^2^ = 0.01 to η_p_^2^ = 0.058 (*d* = 0.10–*d* = 0.49); medium: η_p_^2^ = 0.059 to η_p_^2^ = 0.137 (*d* = 0.50–*d* = 0.79); and large: η_p_^2^ ≥ 0.138 (*d* ≥ 0.80).

## 3. Results

Table 1 shows the descriptive statistics and the Pearson correlations between the variables. The correlation matrix shows that, with the exception of the correlation between claimed self-handicapping and defensive pessimism, all correlations are statistically significant (*p* < 0.001). From a statistical perspective, the results provided by Bartlett’s sphericity test show that the variables are sufficiently intercorrelated (χ^2^(6) = 820.44; *p* < 0.001), a critical condition for subsequent multivariate analyses. Likewise, the asymmetry and kurtosis data indicate that the variables have a normal distribution.

### 3.1. Identification of Profiles of Self-Esteem and Achievement Goals

The adjustment of several models of latent profiles (models from two to six classes) has been analyzed. The models were adjusted assuming that the variances could differ between the indicators within each group, but it was specified as a restriction that they were equal between the groups. Likewise, the independence between the indicators was imposed as a restriction, that is, within each group and between groups.

Table 2 shows the results of the model adjustment. The adjustment of the six-class models was stopped for several reasons: (a) The values of the AIC, BIC, and SSA-BIC statistics were higher in the model of six classes than in the model of five; (b) the LRT and the PBLRT of the six-class model were not statistically significant (*p* > 0.05, in both cases), which indicates that the six-class model does not have a better fit than the five-class model; and (c) in the six-class model, a group of participants with a representation of less than 5% of the total sample was obtained, and in the five-class model, all groups exceeded that percentage. Only the entropy value and the multivariate asymmetry and kurtosis tests suggested that both models presented a similar model fit.

Although, as a whole, the statistical data supported a better fit of the five-class model compared with that of the six-class model, when compared with the four-class model, we observed that the five-class model had a similar but slightly lower AIC, BIC, and SSA-BIC values than the four-class model. The LRT and the PBLRT showed statistically significant values in both models. However, the entropy value was higher in the four-class model (0.973) than in the five-class model (0.928), which indicates a lower classifying accuracy of the latter. Additionally, when comparing in both models the a posteriori coefficients of probabilities of each subject belonging to a given class, we observed that the coefficients of the four-class model were closer to 100%, indicative of a very high classifying accuracy. Table 3 shows the classifying accuracy of the four-class model and the number of subjects (total sample and by sex) that comprise each class, that is, in absolute (*n*) and relative (%) terms. Each row of Table 3 considers the coefficients of the a posteriori probabilities of each subject belonging to a given class. The coefficients associated with the groups to which the participants have been assigned are shown along the principal diagonal of the table.

Likewise, the MANOVA results showed statistically significant differences among the four classes in the criterion variables: Self-esteem (*F*(3,1024) = 4370.71; *p* < 0.001; η_p_^2^ = 0.928), learning goals (*F*(3,1024) = 835.31; *p* < 0.001; η_p_^2^ = 0.710), performance avoidance goals (*F*(3,1024) = 219.02; *p* < 0.001; η_p_^2^ = 0.391), and performance approach goals (*F*(3,1023) = 534.88; *p* < 0.001; η_p_^2^ = 0.610). The effect size was large in all cases. Additionally, in the four-class model and the five-class model, the composition of each of the resulting profiles was analyzed based on conceptual criteria, demonstrating that in the five-class model, two practically identical motivational profiles were obtained. Therefore, the four-class model was better suited to the principle of parsimony that should govern the choice of conglomerates [81].

In summary, based on the statistical data related to the adjustment of models, according to the results of the MANOVA conducted to analyze the contribution of each of the variables making up the profiles to the ability of differentiating between classes, and also according to the conceptual criteria, the four-class model was the most appropriate.

### 3.2. Description of Profiles of Self-Esteem and Achievement Goals

The average scores of the subjects belonging to the latent classes of the chosen model are presented in Table 4.

**Figure 2:**
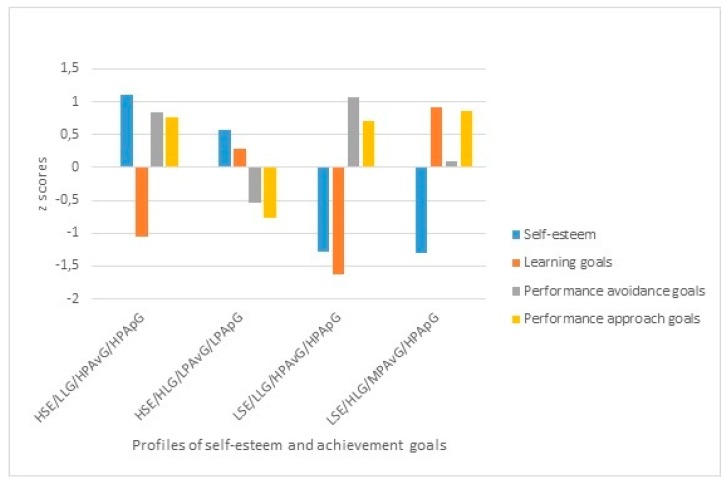
Graphical representation of profiles of self-esteem and achievement goals. Note. HSE/LLG/HPAvG/HPApG: Profile of high self-esteem, low learning goals, high performance avoidance goals, and high performance approach goals; HSE/HLG/LPAvG/LPApG: Profile of high self-esteem, high learning goals, low performance avoidance goals, and low performance approach goals; LSE/LLG/HPAvG/HPApG: Profile of low self-esteem, low learning goals, high performance avoidance goals, and high performance approach goals; LSE/HLG/MPAvG/HPApG: Profile of low self-esteem, high learning goals, medium performance avoidance goals, and high performance approach goals.

Two profiles with high self-esteem and two profiles with low self-esteem were obtained, each of these profiles differed in the level of use of achievement goals. Thus, one group (*n* = 153; 14.88%) could be characterized by presenting high self-esteem combined with a low orientation to learning goals and a high orientation to performance goals (avoidance and approach) . The second group (*n* = 520; 50.58%) also presents high self-esteem, although it is combined with a high orientation to learning goals and a low orientation to performance avoidance and performance approach goals. The third group (*n* = 124; 12.07%) presents low self-esteem combined with a low use of learning goals and a high orientation to performance avoidance and performance approach goals . The fourth group (*n* = 231; 22.47%) comprises participants with low self-esteem and a high orientation to learning goals, moderate performance avoidance goals, and high performance approach goals. The graphic representation of these profiles is shown in Figure 2.

### 3.3. Relationship between Profiles of Self-Esteem/Achievement Goals and Self-Protection Strategies

At the multivariate level, the profiles of self-esteem and achievement goals and the three self-protection strategies are significantly related (λ_Wilks_ = 0.348, *F*(9,2487) = 149.91, *p* < 0.001, η_p_^2^ = 0.296). At the univariate level, the profiles are significantly associated with the three external variables as follows: Behavioral self-handicapping (*F*(3,1024) = 111.18, *p* < 0.001, η_p_^2^ = 0.246), claimed self-handicapping (*F*(3,1024) = 93.87, *p* < 0.001, η_p_^2^ = 0.216), and defensive pessimism (*F*(3,1024) = 341.52, *p* < 0.001, η_p_^2^ = 0.500). The effect size is large in all three cases.

Table 5 shows the descriptive statistics obtained from the MANOVA. As shown, in the cases of behavioral self-handicapping and claimed self-handicapping, the profile tending to significantly use both strategies is that which combines low self-esteem with low learning goals, high performance avoidance goals, and high performance approach goals. The post hoc contrasts (Games–Howell) reveal that the differences with respect to the other profiles are, in addition to being statistically significant, large (between *d* = 1.51 and *d* = 1.86) in all cases. The profile characterized by low self-esteem, low learning goals, high performance avoidance goals, and high performance approach goals also shows a significantly higher use of defensive pessimism than the two profiles with high self-esteem (i.e., the profile that shows high self-esteem, low self-esteem, high performance avoidance goals, and high performance approach goals; and the profile with high self-esteem, high learning goals, low performance avoidance goals, and low performance approach goals), with medium effect sizes (*d* = 0.73 and *d* = 0.69, respectively). By contrast, the profile characterized by low self-esteem, high learning goals, medium levels of performance avoidance goals, and high performance approach goals was significantly more related to defensive pessimism, demonstrating large differences with respect to the three remaining profiles (between *d* = 1.75 and *d* = 2.47).

Considering the scores obtained by men and women of each motivational profile in the three self-protection strategies, it was observed that in the profile of low self-esteem, low learning goals, high performance avoidance goals, and high performance approach goals, men significantly used behavioral self-handicapping to a greater extent (χ^2^(23) = 36.98, *p* < 0.05), whereas women significantly used claimed self-handicapping to a greater extent (χ^2^(29) = 50.82, *p* < 0.05). On the contrary, in the profile of low self-esteem, high learning goals, medium performance avoidance goals, and high performance approach goals, claimed self-handicapping was significantly more used by men (χ^2^(32) = 56.83, *p* < 0.05).

## 4. Discussion

Researchers of education are increasingly aware that students diverge not only in cognitive aspects, but also in motivational aspects. Adopting a person-centered approach, this study aimed to analyze the formation of different motivational profiles from the combination of self-esteem and achievement goals. Likewise, we intended to identify profiles that underlie the use of self-protection strategies of personal worth.

Regarding the first objective, four motivational profiles have been differentiated: Two with high self-esteem and two with low self-esteem. The four profiles differ from one another in the achievement goals they adopt. On the one hand, as hypothesized, our data suggest the existence of a large group of students characterized by high self-esteem and high learning goals but with little concern for performance goals (i.e., avoidance and approach). These students, therefore, show feelings of appreciation and value to themselves, and they are academically oriented to improve their knowledge without caring about the social comparison. Consequently, they are students neither motivated by a desire to show their superiority to classmates, teachers, or parents, nor worried about receiving negative criticism for their academic competence. This profile (i.e., high self-esteem, high learning goals, low performance avoidance goals, low performance approach goals) seems to fit with the characteristics of the “success-oriented” profile [82]. Success-oriented students are typically self-confident students who focus their efforts on learning and perfecting themselves as students, without fearing poor performance or questioning their personal worth. In effect, the multiple goal literature has endorsed the adaptiveness of this profile because it has been positively related to high levels of engagement, performance, and emotional wellbeing [64].

Additionally, although not exactly as expected, we observed evidence of a second profile of students with high self-esteem. Contrary to the profile of high learning goals and low performance goals (avoidance and approach), this second profile of students with high self-esteem has eminently focused on performance goals (in their tendencies towards avoidance and approach), but not on learning goals. Therefore, they are oriented toward demonstrating their competence, but not to increase it [34]. In other words, the students who are salient in performance goals judge their academic competence based on interpersonal standards, such that they move between the desire to be praised for their abilities and the fear of acquiring a negative social image. That finding may indicate, as Dweck and Leggett [83] suggested, that when students pursuing performance approach goals experience some fails, they could display doubts about their ability to be evaluated as better than others and redirect their priorities to focus on performance avoidance goals.

Regarding the profiles with low self-esteem, as we hypothesized, there exists a profile of students with low learning goals, high performance avoidance, and high performance approach goals. Thus, this students show the same pattern of achievement goals that the students characterized in the previous paragraph, differing only in their level of self-esteem. A priori, the identification of a profile of students with low learning goals and a high adoption of performance goals (i.e., avoidance and approach) associated with low self-esteem is not surprising, given that the latter usually implies a high emotional vulnerability to criticism and an excessive desire to gain social approval [84], aspects that also define performance goals. However, our data do not allow us to offer an accurate answer to why some students with low learning goals and high performance goals (both avoidance and approach) show low self-esteem and other students with the same achievement goals profile show high self-esteem. A plausible assertion is that differences in self-esteem are associated with the level of performance achieved. Previous research has shown that among students who adopt high performance goals, there is a high fear of failure, associated with the connection established by these students between self-worth and their need to demonstrate their competence [3]. Thus, attaining high performance would lead to a competitive social image and, with it, high self-esteem. By contrast, low performance would lead to a less competitive social image and, therefore, low self-esteem. In any case, this is merely a tentative explanation that should be analyzed more rigorously by future research.

Finally, and also as expected, we observed a second profile of students with low self-esteem. In terms of achievement goals, this student profile combines a high interest in learning (i.e., learning goals) with a moderate concern over presenting a social image of incompetence (performance avoidance goals) and a strong desire to stand out and be considered a high performer (performance approach goals). In line with this finding, some works on multiple goals in university students [49,61] have observed a profile that combines the adoption of learning goals with both types of performance goals in a high degree. As stated by Wormington and Linnenbrick-García [64], this student profile has generally been considered equally as adaptive as the profile of high learning goals and low performance goals (i.e., avoidance and approach) in terms of engagement and performance. However, less beneficial outcomes have been observed regarding control beliefs and emotional wellbeing. Accordingly, the students who conjugate high learning goals with moderate performance avoidance goals and high performance approach goals usually exhibit high academic engagement and performance but vulnerability, for example, anxiety, stress, fear of failure [85], linked to social comparison. The results of our study expand the characterization of this motivational profile by specifying that these students have low self-esteem.

Likewise, our findings suggest that the high use of learning goals is not always associated with high self-esteem. In this sense, Niiya and Crocker [86] asserted that when self-esteem is threatened (i.e., low self-esteem), students may be interested in learning as a means to demonstrate their ability, either in an effort to excel in front of their peers or to avoid appearing incompetent. Therefore, this scenario could be the case of the students that, in our study, embody the profile that combines low self-esteem with high learning goals, medium levels of performance avoidance goals, and high performance approach goals.

In relation to the second objective, the results of this work suggest that two of the identified motivational profiles (the profile characterized by low self-esteem, low learning goals, high performance avoidance goals, and high performance approach goals; and the profile of students who exhibit low self-esteem combined with high learning goals, medium levels of performance avoidance goals, and high performance approach goals) are especially vulnerable to the use of self-handicapping and defensive pessimism, respectively.

This finding may be interpreted in two ways. On the one hand, it seems that, independent of the achievement goals of students, low self-esteem is a risk factor for becoming involved in the self-protection mechanisms of self-worth, with high self-esteem being a protective factor. Thus, our results would be aligned with the findings of other studies that, from a variable-centered approach, link low self-esteem to self-handicapping and defensive pessimism [17,18,22]. On the other hand, our results suggest that beneath the self-handicapping and defensive pessimism are distinct academic motivations. Thus, regarding self-handicapping, the use of this strategy, in its behavioral and claimed forms, is greater in students who, having a negative self-assessment, show a high orientation to both performance goals. This finding would support the consideration of performance avoidance goals as an important determinant of self-handicapping, as suggested by other research [35,40]. However, similar to other studies [37,38], a high use of performance approach goals is also observed in this profile linked to self-handicapping. In this regard, it is possible that some students who seek to excel over other students are also imbued with the need to protect their personal worth when faced with the fear of failing to achieve their goal [4]. For these students, as our results seem to indicate, self-handicapping would become an attractive mechanism of self-protection.

Regarding defensive pessimism, our findings suggest that this strategy is especially recurrent in students with low self-esteem eager to learn and achieve high performance. However, these students also show a moderate motivation to avoid low performance that compromises their personal worth through negative social judgments. This finding is in line with other research [82] that has characterized the defensive pessimist as a student with a high behavioral commitment to success but whose behavior is cognitively linked to the fear of failure.

Additionally, the identification of motivational characteristics that underlie self-protection strategies allows us to corroborate, in accordance with other studies [17,66], that even in students with low self-esteem, the high use of learning goals reduces the likelihood of resorting to self-handicapping. However, our data suggest that this buffering effect would not be achieved with defensive pessimism.

### 4.1. Educational and Health Implications

The findings in this study entail some educational and health implications of scope. Students in the university stage are especially vulnerable to the adoption of self-handicapping and defensive pessimism strategies [36] because of the many academic, social, emotional, and economic challenges they must manage during this period, which may damage their psychological well-being [87,88] in case of failure. In effect, the recurrent use of self-handicapping is associated with important damages, not only for academic performance [7], but also for the student’s psychological health (e.g., decreased self-esteem, social rejection, increased depressive symptoms, reduced satisfaction with life) [5,89,90]. Similarly, the repeated tendency to imagine the worst scenarios—so common among defensive pessimists—may involve long-term costs in the form of emotional and physical problems [12,91]. Other studies have associated defensive pessimism with burnout and, ultimately, with a decrease in academic performance [92].

Considering the dysfunctional nature of self-handicapping and defensive pessimism [8,12], it is, therefore, necessary to eliminate or reduce those factors in the university context that may be especially threatening for profiles of students identified by our study as motivationally more prone to both self-protection strategies. To this end, motivation research [82,93,94,95] has emphasized the importance of educators adopting some guidelines aimed at preventing the use of self-handicapping and defensive pessimism in academic settings: (a) Agree with the students’ learning objectives, activities, or projects to be developed, evaluation criteria, and deadlines; (b) divide the academic tasks and the study into smaller steps and encourage the students to elaborate action plans to carry them out; (c) encourage students to analyze the causes of their mistakes and provide them with additional opportunities to pass exams or improve academic work; (d) emphasize the importance of improving one’s performance and not competing with others, as well as making explicit the positive qualities of each student; (e) provide students with evaluative feedback based on the degree of effort expended and the appropriate/inadequate use of work strategies (i.e., controllable factors); (f) promote cooperative learning structures.

Complementary to these classroom initiatives, there are other therapeutic interventions that have shown to be effective in improving the psychological health of self-handicappers and defensive pessimists, reducing the need to use these strategies. Among these interventions, one could cite the cognitive behavioral coaching (CBC) [96], the motivation and engagement wheel [97], and the self-compassion therapies [98].

### 4.2. Study Limitations

The contributions of this work should be taken with caution, considering the limitations of the study. First, the cross-sectional design adopted did not allow causal relationships between the variables to be extracted. Future works could analyze these types of relations by using longitudinal study designs. Second, the sample used includes only students assigned to two branches of knowledge (educational and health), which restricts the possible generalization of the results to the whole university population. Third, more than 86% of the participants in the sample were women, which could have influenced the findings. In this regard, it has been observed that in the present study, women got involved in the four motivational profiles by a wide margin. However, the percentage of men is almost double in the profile of low self-esteem, low learning goals, high performance avoidance goals, and high performance approach goals than in the three remaining profiles. Therefore, future studies that have more symmetrical samples regarding gender should be conducted to observe the extent to which these differences between women and men are replicable in terms of the motivational profiles they adopt. Likewise, other studies should contemplate the gender perspective to analyze the relationship between motivational profiles and strategies of self-protection. In a tentative way, our data suggest the possible existence of statistically significant differences between women and men in the use of claimed self-handicapping (both in the profile of low self-esteem, low learning goals, high performance avoidance goals, and high performance approach goals, and in the profile of low self-esteem, high learning goals, medium performance avoidance goals, and high performance approach goals) and behavioral self-handicapping (profile of low self-esteem, low learning goals, high performance avoidance goals, and high performance approach goals). Considering the important imbalance in the representation of both sexes in our sample, future research should analyze with greater rigor the differences observed in the present study. Fourth, the data for this study have been gathered from self-report tests. Future work could benefit from the wealth of information provided by a combination of methods that include classroom observations, questionnaires, and interviews with students. Finally, the goals and self-protection strategies used in this study are not the only ones that students can adopt, and this factor should be considered. In further research, therefore, the existence of motivational profiles should be analyzed from the inclusion of other goals, namely, academic or social. In this regard, the inclusion of other goals, such as work avoidance goals, might allow differences in the use of claimed and behavioral self-handicapping to be observed, as stated by other studies [38]. Likewise, other self-protection strategies, such as self-affirmation, could be considered.

## 5. Conclusions

The results of this work contribute to broadening existing knowledge about the complex motivational reality of university classrooms [99] by determining the existence of unprecedented profiles of students according to how they integrate two basic elements of academic motivation, namely, achievement goals and self-esteem. Specifically, our results allow us to improve the characterization of the profiles of the multiple goals identified in the literature. Thus, those students with high learning goals and low performance goals (i.e., avoidance and approach) are also characterized by their high self-esteem, and students who adopt the three goals to a high degree (or moderately high, in the case of performance avoidance goals) exhibit low self-esteem. Our data also allow us to determine the existence of two differentiated profiles of students with low learning goals and high-performance goals (i.e., avoidance and approach): High self-esteem and low self-esteem. Likewise, the identification of motivational profiles particularly linked to self-handicapping (profile of low self-esteem, low learning goals, high performance avoidance goals, and high performance approach goals) and defensive pessimism (profile of low self-esteem, high learning goals, medium performance avoidance goals, and high performance approach goals) allows for an effective response not only to the question of what factors determine the adoption of these strategies but also to the question of who is more vulnerable to these factors.

## Figures and Tables

**Table 1:** Descriptions and correlations between self-esteem, achievement goals, and self-protection strategies (*N* = 1028).

	1	2	3	4	5	6	7
1. SE	−						
2. LG	−0.12 *	−					
3. PApG	−0.46 *	−0.37 *	−				
4. PAvG	−0.22 *	−0.18 *	0.56 *	−			
5. BSH	−0.31 *	−0.31 *	0.21 *	0.09 *	−		
6. CSH	−0.16 *	−0.31 *	0.22 *	0.09 *	0.64 *	−	
7. DP	−0.59 *	0.28 *	0.11 *	0.43 *	0.09 *	−0.01	−
*M*	3.41	3.24	3.24	3.30	2.04	1.94	2.35
*SD*	0.52	1.00	0.87	0.93	0.77	0.76	0.87
Asymmetry	−0.39	−0.45	−0.60	−0.51	0.96	0.88	0.83
Kurtosis	−1.41	−0.65	0.05	−0.61	−0.05	−0.27	−0.49

Note: SE = self-esteem; LG = learning goals; PAvG = performance avoidance goals; PApG = performance approach goals; BSH = behavioral self-handicapping; CSH = claimed self-handicapping; DP = defensive pessimism. All measurement scales range from 1 to 5, where the highest scores reflect a higher level of self-esteem, achievement goals and self-protection strategies; * *p* < 0.001.

**Table 2:** Statistics for identifying model adjustment of latent classes and classifying accuracy.

	Models of Profiles of Self-Esteem and Achievement Goals				
	Two Classes	Three Classes	Four Classes	Five Classes	Six Classes
AIC	8395.682	7625.779	6953.791	6668.335	6678.335
BIC	8459.842	7714616	7067.304	6806.526	6841.202
SSA-BIC	8418.552	7657.446	6994254	6717.595	6736.391
Entropy	0.999	0.949	0.973	0.928	0.935
Number of groups with *n* ≤ 5%	0	0	0	0	1
LMRT	1471.051 **	758.042 **	1069.317 **	287.174 *	0.000
PBLRT	1513.473 **	779.902 **	1100.153 **	295.455 **	0.000
Multivariate asymmetric adjustment test	0.000	0.000	0.020	0.310	0.320
Multivariate kurtosis adjustment test	0.160	0.650	0.030	0.320	0.250

Note: The models were adjusted assuming that the variances could differ between the indicators within each group, but it was specified as a restriction that they were equal between the groups. Likewise, the independence between the indicators was imposed as a restriction, both within each group and between groups. AIC = Akaike information criterion; BIC = Schwarz Bayesian information criterion; SSA-BIC = BIC adjusted for the sample size; LMRT = adjusted Lo–Mendell–Rubin maximum likelihood ratio test; PBLRT = parametric bootstrap likelihood ratio test; * *p* < 0.01; ** *p* < 0.001

**Table 3:** Characterization of the latent profiles and classifying accuracy of the individuals in each profile.

	Latent Profiles				*n* (%)	*n*_gender_ (%)	
	1	2	3	4		Female	Male
1. HSE/LLG/HPAvG/HPApG	**0.967**	0.033	0.000	0.000	153 (14.88)	136 (88.9)	17 (11.1)
2. HSE/HLG/LPAvG/LPApG	0.015	**0.985**	0.000	0.000	520 (50.58)	449 (86.4)	71 (13.6)
3. LSE/LLG/HPAvG/HPApG	0.000	0.000	**0.991**	0.009	124 (12.07)	95 (76.6)	29 (23.4)
4. LSE/HLG/MPAvG/HPApG	0.000	0.000	0.003	**0.997**	251 (22.47)	207 (89.6)	24 (10.4)

Note: HSE/LLG/HPAvG/HPApG: Profile of high self-esteem, low learning goals, high performance avoidance goals, and high performance approach goals; HSE/HLG/LPAvG/LPApG: Profile of high self-esteem, high learning goals, low performance avoidance goals, and low performance approach goals; LSE/LLG/HPAvG/HPApG: Profile of low self-esteem, low learning goals, high performance avoidance goals, and high performance approach goals; LSE/HLG/MPAvG/HPApG: Profile of low self-esteem, high learning goals, medium performance avoidance goals, and high performance approach goals. The coefficients associated with the groups to which the participants have been assigned are shown in bold.

**Table 4:** Description of latent profiles (means, standard errors, and confidence intervals).

	*M*	*SE*	Confidence Intervals	
			Lower 5%	Upper 5%
HSE/LLG/HPAvG/HPApG (*n* = 153)				
Self-esteem	3.99	0.02	3.96	4.01
Learning goals	2.21	0.06	2.11	2.28
Performance avoidance goals	3.94	0.04	3.87	4.08
Performance approach goals	3.98	0.03	3.92	4.10
HSE/HLG/LPAvG/LPApG (*n* = 520)				
Self-esteem	3.71	0.01	3.69	3.72
Learning goals	3.54	0.03	3.49	3.58
Performance avoidance goals	2.78	0.04	2.71	2.83
Performance approach goals	2.59	0.03	2.53	2.63
LSE/LLG/HPAvG/HPApG (*n* = 124)				
Self-esteem	2.75	0.01	2.72	2.77
Learning goals	1.62	0.03	1.52	1.72
Performance avoidance goals	4.17	0.03	4.05	4.29
Performance approach goals	3.96	0.03	3.86	4.07
LSE/HLG/MPAvG/HPApG (*n* = 231)				
Self-esteem	2.74	0.01	2.72	2.76
Learning goals	4.15	0.03	4.09	4.23
Performance avoidance goals	3.33	0.03	3.24	3.42
Performance approach goals	4.10	0.04	4.02	4.17

Note: HSE/LLG/HPAvG/HPApG: Profile of high self-esteem, low learning goals, high performance avoidance goals, and high performance approach goals; HSE/HLG/LPAvG/LPApG: Profile of high self-esteem, high learning goals, low performance avoidance goals, and low performance approach goals; LSE/LLG/HPAvG/HPApG: Profile of low self-esteem, low learning goals, high performance avoidance goals, and high performance approach goals; LSE/HLG/MPAvG/HPApG: Profile of low self-esteem, high learning goals, medium performance avoidance goals, and high performance approach goals. All measurement scales range from 1 to 5, where the highest scores reflect a higher level of self-esteem and achievement goals.

**Table 5:** Descriptive statistics (means and standard deviations) corresponding to profiles of self-esteem and achievement goals in self-handicapping and defensive pessimism.

Profiles of Self-Esteem and Achievement Goals		BSH	CSH	DP
		*M* (*SD*)	*M* (*SD*)	*M* (*SD*)
1. HSE/LLG/HPAvG/HPApG	Females	1.89 (0.59)	1.84 (0.66)	1.94 (0.50)
	Males	1.95 (0.47)	1.94 (0.71)	1.91 (0.44)
	Total	1.90 (0.57)	1.85 (0.66)	1.94 (0.50)
2. HSE/HLG/LPAvG/LPApG	Females	1.96 (0.65)	1.85 (0.62)	1.97 (0.50)
	Males	1.82 (0.57)	1.77 (0.62)	1.94 (0.51)
	Total	1.94 (0.64)	1.84 (0.62)	1.96 (0.50)
3. LSE/LLG/HPAvG/HPApG	Females	2.97 (1.05)	3.12 (0.93)	2.47 (1.11)
	Males	3.36 (0.69)	2.11 (1.03)	2.11 (0.67)
	Total	3.06 (0.99)	2.88 (1.05)	2.39 (1.04)
4. LSE/HLG/MPAvG/HPApG	Females	1.81 (0.55)	1.68 (0.53)	3.46 (0.62)
	Males	1.91 (0.63)	1.87 (0.80)	3.44 (0.66)
	Total	1.82 (0.56)	1.70 (0.56)	3.46 (0.62)

Note. HSE/LLG/HPAvG/HPApG: Profile of high self-esteem, low learning goals, high performance avoidance goals, and high performance approach goals; HSE/HLG/LPAvG/LPApG: Profile of high self-esteem, high learning goals, low performance avoidance goals, and low performance approach goals; LSE/LLG/HPAvG/HPApG: Profile of low self-esteem, low learning goals, high performance avoidance goals, and high performance approach goals; LSE/HLG/MPAvG/HPApG: profile of low self-esteem, high learning goals, medium performance avoidance goals, and high performance approach goals; BSH = behavioral self-handicapping; CSH = claimed self-handicapping; DP = defensive pessimism; All measurement scales range from 1 to 5, where the highest scores reflect the highest level of self-esteem, achievement goals and self-protection strategies.
